# What do future physicians think of traditional, complementary, and integrative medicine (TCIM)? Fifteen years after the inclusion of TCIM in the Swiss constitution

**DOI:** 10.1371/journal.pone.0333920

**Published:** 2025-12-03

**Authors:** Ilana Berlowitz, Helena Adler, Daniel Gallego Perez, Arnoud J. Templeton, Ursula Wolf

**Affiliations:** 1 Institute of Complementary and Integrative Medicine, Faculty of Medicine, University of Bern, Bern, Switzerland; 2 Faculty of Medicine, University of Basel, Basel, Switzerland; 3 Department of Physical Medicine & Rehabilitation Program on Integrative Medicine, University of North Carolina at Chapel Hill, United States of America; 4 Department of Oncology, St. Claraspital, Basel, Switzerland; 5 St. Clara Research, Basel, Switzerland; Endeavour College of Natural Health, AUSTRALIA

## Abstract

**Background:**

An increasing recognition of traditional, complementary and integrative medicines’ (TCIM) contributions for public health has driven the development of international policy recommendations for its inclusion into national healthcare systems. While many countries have made advances in the incorporation of TCIM in academic medicine, there are globally only a handful that have a constitutional mandate in this context. The present cross-sectional study focused on one of these, namely Switzerland, some fifteen years after TCIM was included into the country’s Federal Constitution.

**Aims & methods:**

This research aimed to examine attitudes of the future medical workforce in regard to TCIM within the only European country in which TCIM is mandated by the constitution. More specifically, using an online survey tailored to the country’s socio-political context, this study assessed attitudes, knowledge, and expectancies regarding TCIM among medical students across all Swiss universities that offer degrees in human medicine.

**Results:**

Even though Swiss medical schools have consolidated and expanded their TCIM-related curricula compared to earlier assessments, with all of them now offering mandatory TCIM classes, most participants were unaware of this. Nonetheless, two-thirds of the *N* = 695 participants considered TCIM a valuable extension of conventional medicine that should have a place in medical education. Views diverged greatly between TCIM modalities. Further, we found significant differences as a function of gender and linguistic regions, although in the latter case effect sizes were modest.

**Conclusions:**

Knowing the views of medical students as the future generation of physicians, clinical scientists, and often also decision-makers in the context of policy-driven integration is crucial in understanding the future trajectory of TCIM into mainstream healthcare. Based on Switzerland’s unique experience and history in this context this work contributes to the broader discourse on the role of TCIM in national healthcare systems.

## Introduction

The last decades’ growing interest in the role of traditional, complementary, and integrative medicines (TCIM) in human health and wellbeing has led to the development of international policy recommendations for TCIM’s integration into national healthcare systems (Wold Health Organization (WHO) [1,2]), based on the increasing recognition of TCIM’s advantages for health access (accessibility, affordability, acceptability [3]) and towards achieving the Sustainable Development Goals of Universal Health Coverage (UHC [4]). According to the WHO webpage [5] traditional medicine is defined as “codified or non-codified systems for healthcare and well-being, comprising practices, skills, knowledge and philosophies originating in different historical and cultural contexts, which are distinct from and pre-date biomedicine, evolving with science for current use from an experience-based origin”; complementary medicine is defined as “additional healthcare practices that are not part of a country’s mainstream medicine”, and integrative medicine is defined as “an interdisciplinary and evidence-based approach to health and well-being by using a combination of biomedical and traditional and/or complementary medical knowledge, skills and practices”. To support its recommendations, the WHO has stepped up actions in this context, introducing a TCIM chapter to the 11^th^ revision of the International Classification of Diseases (ICD-11) [6], creating the Global WHO Centre for Traditional Medicine to advance research and policy developments (Headquarters in Jamnagar, India), and convened the first global TCIM summit, in which a call for a wide-range action agenda toward health and well-being of people and the planet was formulated (‘Gujarat Declaration’) [7].

Following the WHO recommendations, international advances exploring TCIM’s contributions in healthcare systems toward UHC have been increasing [e.g., 8, 9]. Indeed, in their most recent global survey, the WHO reported TCIM use in all countries that participated in the survey (88% of Member States) [WHO, 2]. Nonetheless, the report also showed that only 26% of participant countries included some type of insurance coverage (be it public or private) for TCIM treatments, indicating that, *de facto*, integration of TCIM methods into healthcare systems is still uncommon. Indeed, although most countries likely have some form of TCIM policy, law, or regulation [WHO, 2, 10], worldwide there are only a handful, among them Switzerland, which have enshrined a mandate to consider these therapeutic modalities within their Constitution [WHO, 2, 11].

A constitution is defined as the “system or body of fundamental principles according to which a nation, state, or body politic is constituted and governed” [12]; it thus represents the highest form of political mandate in a given country. In Switzerland, a referendum (a so-called counter-proposal to a ‘federal popular initiative’) proposing a new constitutional article regarding TCIM was endorsed by a population majority and all cantons in 2009 [13], resulting in the addition of Article 118a to the Constitution, which states that the “Confederation and Cantons shall within the scope of their powers ensure that consideration is given to complementary medicine” [14] and which since then provides a strong legal basis for incorporating TCIM into Swiss public healthcare. Switzerland is currently the only European country with a TCIM-related constitutional mandate, hence represents a unique opportunity for examining how such a mandate may lead to manifest changes in availability of TCIM health services, corresponding medical education, or physician attitudes. However, as of today, the extent to which this is the case is not entirely clear. Following the adoption of the constitutional mandate, treatments from four TCIM modalities (namely Traditional Chinese Medicine (TChM), phytotherapy, Anthroposophic Medicine (AM), and homeopathy) were incorporated into the Swiss mandatory (basic) health insurance scheme [15] – first provisionally, and starting from 2017 permanently [15] –, meaning that these treatments are reimbursed in the same fashion as conventional treatments if delivered by physicians certified in one of these modalities [16,17]. The selection of modalities was based on the existence of corresponding accredited training programs (Swiss Institute for Continuing Medical Education [18]) but could in principle be expanded to further modalities. A national level educational policy regarding how TCIM ought to be represented in academic medical education was however still lacking at the time [19] – and still is today.

Nonetheless, the availability of TCIM in public health services seems to have increased; a study prior to the referendum found TCIM services in Swiss hospitals generally low [20], whereas a study focusing on the French-speaking region of Switzerland a few years later observed the number of hospitals offering TCIM to have risen [21]. Similarly for the usage of TCIM services; a periodically conducted national health survey indicated that, although unchanged between 2007 and 2012 [22], Swiss TCIM usage showed a significant increase between 2012 and 2017 [23]. In relation to physician involvement and attitudes, a 2017 survey on 640 Swiss pediatricians found that only 8% had a federal certificate in one of the TCIM methods and the large majority (84%) did not offer TCIM treatments, mainly due to insufficient knowledge, skepticism regarding TCIM methods, and institutional barriers at their workplace [15]. The latter reason was mainly raised by hospital-employed pediatricians, who wanted but were not granted permission to apply TCIM methods.

Indeed, studies have shown that TCIM-related political mandates do not necessarily translate into awareness and acceptance by healthcare workers and managers [24,25]. Research has described barriers at various levels, including systemic obstacles within medical organizations and research institutions, an absence of TCIM education, as well as a lack of knowledge and biased views among healthcare providers [26–29]. The views and attitudes of medical doctors as powerful stakeholders within the health sector –be it in managerial positions that impact the nature and range of available health services, as gatekeepers in patient care, or as key decision makers within clinical trials [30–32]– may be particularly decisive in such translational processes. This paper seeks to explore TCIM-related views among future physicians some 15 years after TCIM was included as a constitutional mandate in Switzerland. More specifically, we aimed to examine TCIM-related attitudes, self-assessed knowledge, perceived effectiveness, expectancies regarding patient views, as well as educational exposure among students attending medical schools in Switzerland. By examining the attitudes of the future medical workforce within the only European country in which TCIM is mandated by the constitution, this research aims to contribute to the broader discourse on the role of TCIM and its future trajectory in healthcare systems.

## Methods

### Setting, data collection, and sampling

This study was conducted by the University of Bern’s Institute of Complementary and Integrative Medicine, Switzerland. Academic studies in human medicine are regulated by the Swiss Confederation and take 6 years in total, divided into bachelor’s and master’s studies (3 years each). At the time of the study a total of 10 Swiss universities were offering degrees in human medicine (bachelor’s and/or master’s), namely the universities in Basel, Bern, Zurich, Fribourg, Geneva, Lausanne, Lucerne, Lugano, St. Gallen, and ETH-Zurich. While it is possible to attend the University of Neuchâtel for the first year of medical studies, this university does not offer a degree in human medicine and was hence not considered in the current study. We contacted all medical faculties via email to request detailed information regarding their TCIM-related curricular activities and consulted the respective websites. After compiling a summary of the information obtained, we sent the respective excerpt of the curricular overview to each university for confirmation.

To prepare the online survey we used *EFS Survey* by *Unipark/Tivian* (Tivian XI GmbH). We employed a convenience sampling approach to reach as many eligible subjects as possible [33,34]. Inclusion criteria involved being enrolled at one of the above medical schools (bachelor’s or master’s degree), while individuals who provided incomplete survey data were excluded from the study. Online access to the survey was provided via a non-personalized link, sent out to prospective participants through the Swiss Medical Students Association (Swimsa [35]) and the Medical Students Councils of the various Swiss universities in October 2022. A reminder was sent two months later, and data collection was closed by the end of December 2022. Prospective participants were thoroughly informed about the study objectives, and that participation was voluntary and anonymous. Those interested to take part provided implied consent via opting in. The responsible ethics committee (Canton of Basel–*Ethikkommission Nordwest- und Zentralschweiz*) waived the need for ethics approval given full anonymity of participants in the online survey.

### Instrument

To assess medical students’ views and attitudes toward TCIM we designed a survey instrument tailored to the Swiss academic and socio-political context. The survey was pilot tested in September 2022 using a pilot sample of 23 medical students across Swiss universities, which led to minor adaptations in the survey, namely the wording of some items was slightly adjusted to improve clarity and correct errors, and two non-essential items were removed from the survey to keep the survey completion time as brief as possible. The final survey comprised 32 items covering the following topics: personal overall attitudes towards TCIM (*In my opinion TCIM is a valuable extension of conventional medicine and should have a place in medical education*), general interest in TCIM (*I am generally interested in TCIM methods and its applications*), opinions about TCIM-related curricula (*In my opinion medical students learn enough about …*), impressions regarding patient views *(I think patients are interested in …; I think patients find … beneficial*), clinical effectiveness (*In my opinion, … has shown clinical effects in treating patients*), intention to apply TCIM methods in their future clinical practice *(I may consider using … to treat patients*), personal past use (*Have you personally used* …), and self-assessed degree of knowledge (*I am confident that I can provide patients with basic information about...*) on the four TCIM methods. The items were presented as statements and rated on a 5-point Likert scale ranging from 1 (*strongly agree*) to 5 (*strongly disagree*), except for the ones assessing past personal use of TCIM methods and if TCIM was being taught at their university (response format *yes*/*no*/*I don’t know*). The questionnaire can be obtained from the authors upon request. We additionally assessed gender, year of studies, and academic institution as basic sample characteristics. Given the linguistic variety of Switzerland, the survey was provided in English, a language in which the target population could be expected to be fluent enough to complete the survey.

### Data analysis

We used *SPSS Statistics* Version 29.0 [36] for data analysis, employing descriptive statistics to report demographic sample data and the distribution of attitudinal items, and tested for subgroups differences in regard to sex, academic year, and linguistic regions (grouping based on location of affiliated university, namely Swiss German-speaking region: Zurich/ETH, Basel, Bern, Lucerne, St. Gallen; Swiss French-speaking region: Lausanne, Fribourg, Geneva; and Swiss Italian-speaking region: Lugano) on (a) overall attitude towards TCIM and (b) general interest in TCIM. Overall attitude and general interest each represented single-item measures and were calculated as mean scores on the corresponding item (see section *Instrument*). As both dependent variables significantly departed from normality (Shapiro-Wilk test: overall attitude towards TCIM – *W*(695) =.88, *p* < .001; general interest in TCIM – *W*(695) =.90, *p* < .001) we opted for the nonparametric Kruskal-Wallis (*H*) tests (*α *< 0.05). In case of significant *H*-tests we performed post-hoc analyses for pairwise comparisons using Dunn’s test with Bonferroni (BF)-adjusted *p* levels, also providing Cohen’s *r* [37] as an effect size measure in case of significance. Finally, Spearman’s rank-order correlation was used to assess the bivariate association between the dependent variables. An a priori power analysis using G*Power [38] version 3.1.9.7 with α = .05 and power = 0.95 showed that the minimum sample size needed for H-tests to detect medium effect sizes (0.25) is *N* = 210 for 2 groups (sex), *N* = 305 for 3 groups (language region), and *N* = 324 for 6 groups (academic year).

## Results

### TCIM-related content in medical school curricula

An overview of curricular activities related to TCIM in Swiss academic institutions offering medical degrees is shown in Table 1. Although we have received information on curricular activities from the University of Lausanne, confirming that they offer TCIM-related courses and that some of them are mandatory, they eventually preferred not to exhibit any detailed information on their curriculum here. Overall, all of the 10 medical schools provide TCIM-related courses (lectures, seminars, and other types of teaching) covering different topics, with a tendency to focus on general TCIM foundations as well as basic principles of specific TCIM modalities, particularly the four ones included in the mandatory health insurance scheme, as well as practice-related courses. The extent and scope of the courses may vary greatly between medical schools. Nonetheless, all medical schools include mandatory courses related to TCIM.

**Table 1:** Overview of TCIM-related curricular activities at all Swiss universities that offer degrees in human medicine.

	Overall number of TCIM-related courses (and total number of lessons^a^)	Number of mandatory^b^ courses (and total number of mandatory lessons^a^)	Study year/s in which TCIM-related courses taught	Types of courses	Topics of TCIM-related courses
**Bern**	4 (32)	3 (10)	Years 2, 4–6	Lecture courses*; in-depth seminars*; elective practicum; master theses; elective study year	Complementary and integrative medicine, principles of diagnosis and therapies; TCIM in Switzerland (legal aspects, TCIM in the general health care system); different modalities, holistic concept; introduction to anthroposophic medicine; introduction to traditional Chinese medicine; introduction to phytotherapy; introduction to homeopathy; research and scientific base of TCIM; implementation of TCIM in clinical practice and common indications; based on PROFILES learning objectives (all themes are covered in mandatory courses)*
**Geneva**	1 (4)	all	Year 6	Types of courses unknown	Complementary and integrative medicine introduction* (definition; legal, epidemiological, insurance-related bases; complementary medicines in the mandatory health insurance scheme; TCIM and research; hypnosis, mindfulness, scientifically recognized approaches; pharmacotherapy of TChM, acupuncture; classic drugs and phytotherapeutics; homeopathy; anthroposophic medicine phytotherapy; meeting specialists)
**Zurich**	17 (35)	3 (6)	Years 2–6	Lecture courses*, eLearning*, practice lecture course*, group work	Complementary therapies*; foundations of diagnosis and therapy in complementary medicine*; family medicine and the challenge of complementary medicine*; complementary treatments for pain; foundation, application, and evaluation of TCIM treatments – courses on specific TCIM systems and topics such as introduction to TCIM, mind-body medicine, anthroposophic medicine, phytotherapeutic treatments, complementary oncology, naturopathy, introduction to Chinese medicine, critical discussion of scientific state of the art, application of knowledge in consultation, case-related learning evaluation
**Lucerne** ^**c**^					
**St. Gallen** ^**c**^					
**Fribourg**	1 full and 1 partial course (number of lessons unknown)	1 partial course (number of lessons unknown)	Years 3, 6	Lecture course*, optional practicum	Introduction to complementary medicine*, practicum on specific TCIM modalities
**Basel**	10 full and 3 partial courses (ca. 106)	8 (10)	Years 1–5	Lecture courses*, compulsory elective projects*	Introduction to complementary medicine*; Skin – treating external symptoms internally*; analgetic measures in oncology – conventional and complementary medicine*; management of side effects in oncology – conventional and complementary medicine*; acupuncture and hypnosis*; anthroposophic medicine in gynecology, obstetrics, cardiology; integrative medicine*. Partial contribution in: pharmacokinetics, drug interactions*; antibiotics and other therapies for lung infections*
**Lugano** ^**d**^	3 (3)	all	Year 5	Lecture courses*	Complementary medicine*, acupuncture*, psychosomatic medicine*
**ETH Zurich** ^**e**^	2 (5)	all	Years 2–3	Lecture courses*	Skin – treating external symptoms internally*; Complementary medicine*
**Lausanne**	A number of TCIM-related courses, some of which are mandatory, are offered, but they eventually preferred not to exhibit any detailed information here.				

*Note*. TCIM = traditional, complementary, and integrative medicine. ^a^In the Swiss academic context a lesson is equivalent to 45 min. ^b^Mandatory courses are those whose content is included in the assessments (exams); attendance is not necessarily compulsory. ^c^The universities of Lucerne and St.Gallen offer approximately the same TCIM-related courses as the University of Zurich in the context of their joint medical studies program, but minor deviations are possible. ^d^Offers master’s degree studies only. ^e^Offers bachelor’s degree studies only. *Designates mandatory course topics and corresponding course types in case this information is available.

### Sample

A total of 1’245 individuals confirmed reception of the survey invitation by clicking on the survey link in the email. Of these, 765 individuals agreed to participate in the study, but 70 were excluded due to incomplete survey data. The final sample thus consisted of *N* = 695 medical students currently enrolled in Swiss medical schools. In view of the previously described power analysis, the obtained sample size is more than adequate for the analyses performed in this study. About two thirds of the sample identified themselves as female, one third as male (see Table 2). Participants were at varying stages of their medical studies (first to sixth year), attending different medical schools, most commonly the ones of the universities of Lausanne, Basel, or Zurich. Due to an administrative problem the survey link did not reach students from the University of Geneva, hence not represented in the sample. Overall, 62.6% of participants were studying in the German-speaking part of Switzerland, 34% in the French- or French/German bilingual- speaking part, and the remainder in the Italian-speaking part of Switzerland, a rate roughly in line with the general linguistic distribution in Switzerland [39].

**Table 2:** Basic characteristics of the sample.

	*n*	%
Gender		
female	474	68.2
male	212	30.5
non-binary	6	0.9
a gender not listed	1	0.1
prefers not to say	2	0.3
Currently in which year of medical studies		
1. year	118	17.0
2. year	136	19.6
3. year	107	15.4
4. year	157	22.6
5. year	98	14.1
6. year	79	11.4
Medical studies at the university in		
Lausanne	171	24.6
Basel	162	23.3
Zurich	138	19.9
Bern	91	13.1
Fribourg	65	9.3
Lucerne	27	3.9
Lugano	24	3.5
St. Gallen	14	2
ETH Zurich	3	0.4
Does your university teach TCIM?		
yes	290	41.7
no	176	25.3
I don’t know	229	32.9

*Note*. *N* = 695. The survey did not reach students from the university of Geneva due to an administrative error.

**Fig 1:**
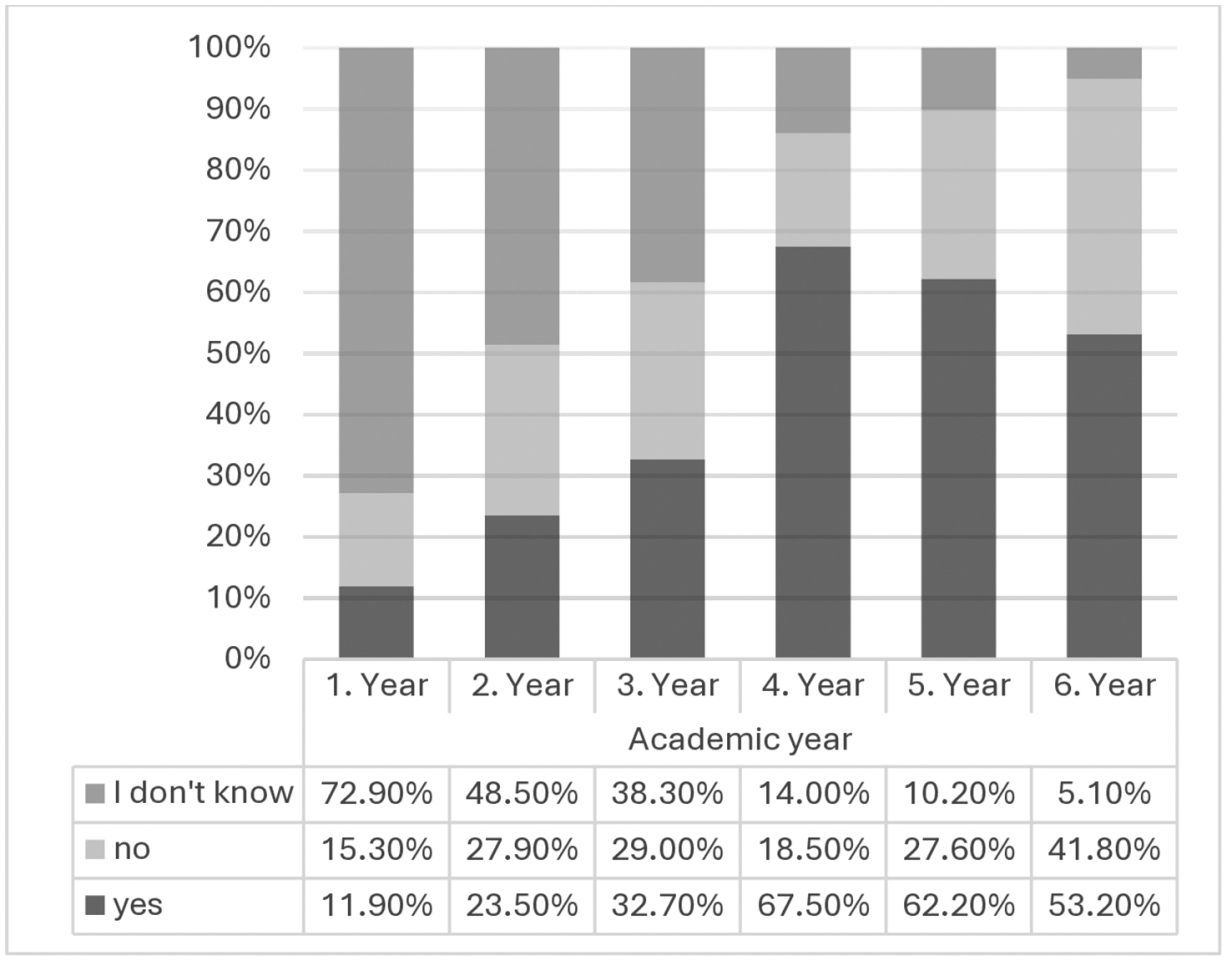
Subjects’ responses to the question if their university teaches TCIM (*N* = 695).

While a third of the respondents (32.9%) were unsure if their university provided TCIM courses and lectures, 41.7% believed theirs did so, and the rest thought their university did not. The proportion of those that were unsure was particularly pronounced in the first three years of study (see Fig 1).

### Attitudes and opinions towards TCIM: descriptive statistics

Table 3 shows the distribution of attitudinal indicators regarding TCIM among participants, both in general as well as in regard to the four specific TCIM modalities. Overall, 60.7% of respondents agreed or strongly agreed that TCIM was a valuable extension of conventional medicine and should have a place in medical education, while 19% disagreed or strongly disagreed with this view. Similarly, 52% of participants expressed general interest in TCIM methods and their application, while 22% disagreed or strongly disagreed. A large majority of participants expected that patients are interested in TChM, homeopathy, and phytotherapy treatments (about 70% each), while a somewhat lower proportion (48.7%) expected patient interest in AM treatments. Similarly, most participants believed that patients generally find TCIM methods beneficial (roughly 70% in case of TChM, phytotherapy, and homeopathy; 54.5% in case of AM), whereas only few disagreed or strongly disagree with this (4–9%). Participants’ ideas regarding clinical effectiveness on the other hand varied between methods: about two-thirds of respondents believed TChM and phytotherapy have shown clinical effects, but only roughly a third did so for AM and homeopathy, and for the latter 38.6% indeed disagreed or strongly disagreed with this opinion.

**Table 3:** Percentages, means, and standard deviations of attitudinal indicators.

			%			*m*	*sd*	*n*
	strongly agree	agree	neutral	disagree	strongly disagree			
General interest in TCIM	17	35	26	13.8	8.2	2.61	1.16	695
Overall attitude of TCIM as a valuable extension of conventional medicine that should be part of curriculum	22.6	38.1	20.1	13.5	5.6	2.41	1.14	695
Medical students learn enough								
… about TChM	23.3	36.5	19.7	13.3	7.3	2.45	1.19	631
… about H	15.7	27.2	22.8	21.3	12.9	2.88	1.27	635
… about PT	21.7	38	20.6	13.2	6.5	2.45	1.16	635
… about AM	19.1	31.7	24.5	15.4	9.2	2.64	1.22	628
Anticipated use of								
…TChM in own clinical practice	5.8	30.4	25.4	20.5	17.8	3.14	1.20	684
… H in own clinical practice	6	26.1	20.5	20.2	27.2	3.37	1.29	687
… PT in own clinical practice	12.7	37.4	27	11.8	11	2.71	1.17	684
… AM in own clinical practice	6.2	21.1	32.8	20.2	19.7	3.26	1.18	674
Patients are interested in								
… TChM treatments	9.5	61.5	21.4	6.8	0.7	2.28	0.76	681
… H treatments	13	61.3	19.5	4.8	1.3	2.2	0.77	683
… PT treatments	15.4	54.9	24.8	4.4	0.4	2.2	0.76	681
… AM treatments	5.6	43.1	38.7	10.5	2.1	2.6	0.83	675
This TCIM method has shown clinical effects:								
… TChM	13.6	49.5	21.3	9.1	6.5	2.45	1.05	648
… H	6.3	29.5	25.6	16.8	21.8	3.18	1.25	671
… PT	14.2	47.9	26.5	6.6	4.9	2.4	0.98	635
… AM	7	28.6	38.7	13.5	12.2	2.95	1.09	615
Patients find								
… TChM beneficial	11.8	63	21	3.1	1	2.19	0.72	671
… H beneficial	9.9	58.8	24	4.5	2.8	2.31	0.82	674
… PT beneficial	12.6	53.5	29.2	3.8	0.9	2.27	0.76	665
… AM beneficial	7	47.5	37.2	6.2	2.2	2.49	0.80	646
I can adequately inform patients								
… on TChM	3.7	17.4	16.1	43.3	19.6	3.58	1.10	679
… on H	9.9	30.8	19.9	27.7	11.8	3.01	1.21	679
… on PT	4.3	23.9	21.1	34.9	15.8	3.34	1.13	677
… on AM	3.6	13.1	19.3	38.5	25.5	3.69	1.10	670

*Note.* TCIM = Traditional, complementary, & integrative medicine, TChM = Traditional Chinese Medicine, H = homeopathy, PT = phytotherapy, AM = anthroposophic medicine; mean scores range from 1–5, with higher scores implying less agreement.

About half of respondents anticipated using phytotherapy in their future clinical practice, while roughly a third did so for each of the other modalities, although for homeopathy nearly half of the sample also disagreed/strongly disagreed. Further, as an indicator of their own perceived knowledge on TCIM methods, participants assessed the degree of confidence with which they believed they can provide basic information on TCIM to patients. Overall, except for homeopathy, more participants than not (50.7–64.0%) maintained to have insufficient knowledge to be able to adequately inform patients. Even so, about half of participants considered that medical students learn enough about the four TCIM modalities in medical school.

Overall, nearly three-quarters of the sample reported having experienced at least one of the four TCIM modalities themselves (34.5% reported having used one, 24.7% two, 11.2% three, and 2.7% all four of the TCIM modalities). In terms of the specific modalities, a bit over half of the sample reported having used homeopathy, followed by phytotherapy; somewhat less frequent were experiences with TChM and lastly AM (see Table 4).

**Table 4:** Past personal use of TCIM methods.

		%	
In the past, have you personally used…	yes	no	I don’t know
Traditional Chinese Medicine	22.4	76.70	0.9
Homeopathy	55.4	43.00	1.6
Phytotherapy	40	54.40	5.6
Anthroposophic Medicine	10.8	80.00	9.2

*Note. N* = 695, TCIM=Traditional, complementary, & integrative medicine.

### Subgroup comparisons

Table 5 shows means and standard deviations of the sample’s overall attitude and interest in TCIM within subgroups concerning sex, linguistic regions, and academic study year. *Sex differences:* There was a significant difference in overall attitude between male and female medical students, *H*(1)=105.65, *p* < .001, with female participants expressing a more positive attitude towards TCIM than males. Similarly, female participants also expressed more interest in TCIM compared to males, *H*(1)=95.18, *p* < .001. *Differences by linguistic region:* There was a significant difference between language regions in overall attitude (*H*(2)=33.06, *p* < .001). Post-hoc comparisons showed the difference to be between French- and German-speaking regions (BF-adjusted *p* = .000, *r* = .22), whereas the other pairwise comparisons yielded no significance. Likewise, general interest in TCIM was significantly different between linguistic regions (*H*(2)=33.28, *p* < .001), with post-hoc analysis pointing to significant differences between the Swiss French- vs. German-speaking (BF-adjusted *p* = .000, *r* = .22), as well as French- vs. Italian-speaking (BF-adjusted *p* = .023, *r* = .17) regions. *Differences by academic year:* Participants at different stages in their medical studies did not differ from each other in overall attitude (*H*(5)=6.98, *p* = .22) nor interest (*H*(5)=4.96, *p* = .42).

**Table 5:** Means and standard deviations of the sample across sex, linguistic region, and academic year subgroups.

	f	m	Swiss-GE	Swiss-FR	Swiss-IT	Y1	Y2	Y3	Y4	Y5	Y6
	(*n* = 474)	(*n* = 212)	(*n* = 435)	(*n* = 236)	(*n* = 24)	(*n* = 118)	(*n* = 136)	(*n* = 107)	(*n* = 157)	(*n* = 98)	(*n* = 79)
Overall attitude towards TCIM, *M*(*SD*)	2.09(.97)	3.11(1.18)	2.60(1.16)	2.08(1.04)	2.42(1.10)	2.38(1.11)	2.50(1.08)	2.46(1.16)	2.48(1.11)	2.24(1.20)	2.33(1.27)
−agree or strongly agree, %	71.30	37.20	54.50	73.30	50.00	60.20	54.40	60.80	59.20	70.40	63.20
−disagree or strongly disagree, %	9.90	39.60	24.40	10.20	12.50	16.10	19.10	20.50	20.40	19.40	19.00
General interest in TCIM, *M*(*SD*)	2.31(1.03)	3.28(1.18)	2.79(1.19)	2.26(1.03)	2.88(1.12)	2.65(1.08)	2.60(1.08)	2.76(1.17)	2.64(1.19)	2.44(1.22)	2.53(1.27)
−agree or strongly agree, %	62.70	29.30	45.90	64.90	33.30	46.60	52.90	49.50	53.50	57.10	51.90
−disagree or strongly disagree, %	12.05	42.90	27.8	11.00	25.00	20.30	20.60	25.20	22.90	20.40	22.80

*Note.* TCIM = Traditional, complementary, and integrative medicine; Swiss-GE = Swiss German-speaking region, Swiss-FR = Swiss French-speaking region, Swiss-IT = Swiss Italian-speaking region; mean scores ranged from 1 to 5, higher scores representing less agreement. Overall attitude and general interest in TCIM were significantly correlated (*r*(693) =.750, *p* < .001).

## Discussion

An increasing recognition of the potentials of TCIM for public health has driven the development of international policy recommendations for its inclusion into healthcare systems [WHO, 1, 2]. While many countries have made advances in this context, there are currently only few with a constitutional mandate related to TCIM [2,11,40,41]. The present study focused on the only European one in this context, namely Switzerland, some fifteen years after TCIM had been included into its Federal Constitution. Using an online survey tailored to the country’s relevant policy context, we assessed attitudes, knowledge, and expectancies regarding TCIM among aspiring medical doctors (*N* = 695) across all Swiss universities that offer degrees in human medicine. We additionally inquired with each of the medical faculties about TCIM-related courses within their academic study programs.

Based on the current curricular overview, Swiss medical schools seem to have generally consolidated and expanded their TCIM-related curricula: while our study shows that all schools currently offer mandatory TCIM-related courses, data from 2007 suggests that this was not the case prior to the referendum [19]. Similarly, in a 2004 survey of medical school department directors at universities in Switzerland, Austria, and Germany, only 34% of respondents (31% in Switzerland) indicated that TCIM had already been integrated into the curriculum at their medical schools [42]. Another study around the same time found that only 40% of universities across Europe reported TCIM education in their regular medical curriculum [43]. Indeed, a more recent scoping review concluded that on an international level, TCIM teaching in undergraduate medical education is widely inconsistent and reflects a lack of defined graduate competencies [44]. Clearly, medical education systems are still adapting to the evolving landscape of healthcare that includes TCIM, with great variability between countries. Several countries in which traditional medicine systems hold long-standing historical roots and cultural significance, such as China and India, have establishing medical schools with a fully integrative curriculum, meaning that conventional and TCIM approaches are being taught in equal parts, while for instance in Japan, Korea, and Taiwan a different route was taken, in which specialized university-level medical schools focus on traditional medicine alone [45–47]. Interestingly, although according to our findings all Swiss medical schools currently involve mandatory TCIM classes, not all participants seemed to be aware of this. This could be explained by the fact that newer students at the time of the survey may not yet have attended TCIM classes, which, as per our curricular overview, indeed tend to be provided at the later stages of the study programs (see Table 1), corroborated by the finding that the uncertainty in this context was most pronounced among first- and second-year students. It is also possible that the corresponding information may not be of high enough visibility on the medical schools’ communication platforms.

Overall, the survey results suggest mostly positive or neutral attitudes towards TCIM among medical students. The question if TCIM was considered a valuable extension of conventional medicine and should have a place in medical education was endorsed by about two-thirds of participants in this study, which was slightly below findings of a 2007 survey in Switzerland that found 72.6% of a sample of *N* = 245 sixth-year medical students were in favor of TCIM-related knowledge to be provided at medical schools [48], and 82% considered it a useful supplement to conventional medicine [19]. However, the sample included only medical students in their final year of study and the survey items are not directly comparable to the ones of the current study, both of which could account for the different rates. Nonetheless, in both our and the 2007 survey, opinions diverged greatly between TCIM modalities. For example, in our study nearly twice as many participants believed in clinical effectiveness of TChM and phytotherapy versus homeopathy and AM. The sequence between the four modalities in this context was identical in our and the 2007 survey (TChM, phytotherapy, homeopathy, and lastly AM) [19]. Although our data does not reveal the reasons for the differential views between modalities, it is likely that, beyond their representation in academic education, the ways in which they are discussed in the media and represented in health services play a critical role in shaping opinions. For example, acupuncture, which is a TChM service, is currently more common in Swiss healthcare than AM services and students are thus much more likely to have been exposed to the former compared to the latter, which in turn may impact their opinions. Indeed, participants’ own experience with AM was low in both studies, which may explain relatively lower ratings of AM on other items, although causal directions may not be determined based on our data. Interestingly in both our and the 2007 survey, homeopathy was the one for which participants reported most personal experience and most self-assessed knowledge (capacity to inform patients) relative to the other modalities, despite their relatively low assessment of its effectiveness and anticipated use in their clinical practice. This finding warrants further investigation. Further, the way certain TCIM modalities are presented in the media (e.g., homeopathy [49,50]) may be linked to less positive views among students. In terms of the low perceived capacity to inform patients about TCIM, this could stem from a substantial proportion of respondents not yet having attended TCIM-related courses when completing the survey, as these courses are often provided at later stages of the study program. Moreover, given the mandatory classes are limited in number and duration, it is also understandable that students in general may not feel confident that their knowledge is sufficient to inform patients.

Further, the gender difference we found is consistent with international findings that women generally exhibit more favorable attitudes towards and more frequent use of TCIM services than men, be it among medical students or the general population, both in Switzerland [23] and internationally [51–55]. The reasons for this may be socioculturally complex but should be further investigated. Finally, the differences between linguistic regions in our data suggests aspiring medical doctors in French-speaking Switzerland to hold somewhat more favorable views on TCIM, but the effect sizes were rather modest [37]. Generally speaking, attitudes toward TCIM may be strongly shaped by cultural and psychosocial factors. Although Switzerland is a separate country, its various language regions are known to be culturally close to its corresponding neighboring countries (as they share language, media, workforce, etc.), and the pattern we found indeed aligns with data from a 2023 cross-national survey in Europe, in which TCIM use (and consequently also openness to TCIM) was somewhat higher in France (41.6%) compared to Germany (35.4%) and substantially more so than in Italy (10.6%) [56].

This work has several limitations. First, since participation in the study was voluntary and eligible subjects decided for themselves whether they completed the survey or not, we cannot rule out selection bias. Further, given only about half of the individuals that confirmed reception of the survey agreed to participate and completed the survey in its entirety, nonresponse bias could impacts the generalizability of findings [33], which is however normal in online research [57] and a drawback likely outweighed by the method’s advantages of maximizing sample size and cost-effectiveness. Moreover, recall bias could have impacted the accuracy of responses on the survey item related to own use of TCIM services, although, given the item only required a yes/no answer and no recollection of precise incidents or number of times such services had been used, it is likely that the assessment is relatively accurate. Further, the absence of data from medical students of the University of Geneva may have influenced the national representability of our findings. Yet, given the number of medical students at this institution make up a small fraction of medical students in Switzerland, their absence is unlikely to have altered the findings in a substantial way. Further, given the survey instrument was constructed specifically for the purpose of this study to fit the research question and socio-political context at hand, its face validity is high, but psychometric indicators remain to be established in future research. Future studies should also include items on TCIM modalities not currently covered in the basic health insurance scheme to be able to compare attitudes. Finally, although we used a non-parametric statistical test that can handle unequal sizes of subgroup samples [58–60] and the distribution of participants studying in the various Swiss language regions in our sample roughly reflects these proportions on a population level [61], the number of respondents in the Italian-speaking region was relatively low, which could impact the generalizability of the language region-related subgroup analysis. The current work also has a number of strengths. Given Switzerland’s unique history of TCIM inclusion, based on a constitutional mandate that represents the people’s will, research in this country offers a valuable opportunity to examine how such a mandate may manifest in education and healthcare beliefs. Given medical students represent the future generation of physicians, clinical scientists, and often also decision-makers in the context of policy-driven integration, their perspective is crucial in understanding the future trajectory of TCIM into mainstream healthcare. Further, given the growing demand for TCIM treatments by patient and hence the necessity of future physicians to possess adequate knowledge in this context to advise patients, clarifying students’ awareness in this context and the degree to which TCIM-related subjects are represented in basic medical education is significant. By examining the attitudes of the future medical workforce within the only European country in which TCIM is mandated by the constitution, this work contributes to the broader discourse on the role of TCIM and its future trajectory in healthcare systems.
